# Voltammetric Determination of Isopropylmethylphenols in Herbal Spices

**DOI:** 10.3390/molecules26206095

**Published:** 2021-10-09

**Authors:** Magdalena Jakubczyk, Slawomir Michalkiewicz, Agata Skorupa, Daria Slefarska

**Affiliations:** 1 Institute of Chemistry, Jan Kochanowski University, PL-25406 Kielce, Poland; smich@ujk.edu.pl (S.M.); agata.skorupa@ujk.edu.pl (A.S.); daria.slefarska@gmail.com (D.S.); 2 Institute for Breath Research, University of Innsbruck, A-6850 Dornbirn, Austria

**Keywords:** thymol, carvacrol, acetic acid, microelectrodes, voltammetry, determination

## Abstract

Thymol and carvacrol—the components of herbal spices—are known for their broad biological activity as antimicrobials and antioxidants. For this reason, it is important to develop new methods for their determination in plant material. A simple, rapid, and sensitive method for determination of total content of these analytes in herbal spices using differential pulse voltammetry (DPV) has been developed. The basis of the research is the oxidation process of isopropylmethylphenols on a platinum microelectrode in glacial acetic acid containing acetonitrile (20%, *v*/*v*) and 0.1 mol L^−1^ sodium perchlorate as the supporting electrolyte. Linear voltammetric responses for thymol and carvacrol were obtained in a wide concentration range from 0.39–1105 and 0.47–640 µg mL^−1^, with a low detection limit of 0.04 and 0.05 µg mL^−1^, respectively. The analysis was performed using the multiple standard addition method. The results of the voltammetric determination are in good agreement with the data of the standard chromatographic method. To the best of our knowledge, this is the first presentation of an electrochemical procedure to determine these compounds in these environmental and electrode materials.

## 1. Introduction

**Scheme 1:**
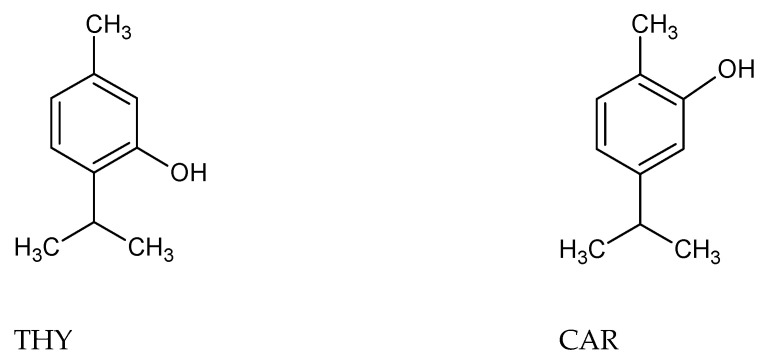
Chemical structures of thymol and carvacrol.

The attractiveness of food is related to its basic organoleptic characteristics, such as taste and smell. In order to strengthen or improve them, additives are used, which are often green plants in the form of dried plants, i.e., spices or essential oils. They improve not only the taste but also the aroma and color of dishes. This, in turn, makes them more attractive and also facilitates the digestion and assimilation of meals. Most herbal spices have additional antioxidant and antibacterial properties and have a positive effect on the digestive, blood, and urinary systems in the human body [1]. The basic ingredients that give plants their characteristic taste and aroma are terpenes. They are most often extracted or distilled from plant material to give essential oils [2,3]. The best known representatives of this class of compounds are isopropylmethylphenols: thymol (THY, 5-methyl-5-(propan-2-yl)phenol) and carvacrol (CAR, 2-methyl-5-(propan-2-yl)phenol). The molecular structures of these compounds are presented in Scheme 1. They are two naturally occurring isomers that belong to the subclass of phenolic monoterpenoids. A rich source of thymol is the essential oil of *Thymus vulgaris* (48.3–78.4%, *w*/*w*), while carvacrol is dominant in the essential oil of *Origanum vulgare* and *Satureja* L. (61.6–87.6% and 43.6–70.7%, *w*/*w*, respectively) [4,5,6,7].

THY affects the functioning of the respiratory system by causing the secretion of mucus in bronchi, which results in an expectorant reflex. More and more attention is paid to its antibacterial, antifungal, antiviral and antiparasitic effects. It also has anti-inflammatory, antiseptic, and acaricidal properties. Moreover, it has an antihyperglycemic effect, accelerates wound healing, and has anticonvulsant and antiepileptic properties [8,9,10]. CAR also has similar therapeutic properties [10]. Thanks to this, thymol and carvacrol are used not only as food additives, but also in the pharmaceutical and cosmetic industries.

Due to the high biological activity of THY and CAR, as well as their widespread occurrence, it is very important to develop methods for their determination in various products. Often used methods are gas chromatography with mass spectrometry (GC-MS) [11,12], or with flame ionization detection (GC-FID) [13,14], and liquid chromatography (LC) [15,16,17,18,19,20,21,22]. In liquid chromatography, spectrophotometric detection in the UV range (RP-HPLC-UV) is used most often. However, colorimetric [23] and spectrophotometric methods [24] are rarely used.

Electrochemical methods, especially voltammetry, can be an alternative to chromatography. In terms of precision, accuracy, limits of detection and quantification, and linearity range, they are comparable to chromatographic techniques (Table 1). Also, they do not require expensive and complex equipment [25].

Voltammetric techniques are based on the anodic oxidation of thymol and carvacrol [26,27,28,29,30,31,32,33,34,35,36,37,38,39,40,41]. From the data described in the literature, it is possible to conclude that the electrode process of THY and CAR on different electrodes and in different solutions is characterized as irreversible [26,27,28,32,33,34,35,37,40,41], controlled by diffusion [27,31,32,33,35,36,39,40,41] or adsorption [34,37], and it involves an exchange of one electron and one proton [30,32,34,35,36,37,40].

The determination of thymol and carvacrol was usually performed on various types of electrode materials, including boron-doped diamond electrode (BDDE) [26,27,28], glassy carbon (GCE) [29,30,31] or modified with CeO_2_ nanoparticle–decorated graphene hybrid film (CeO_2_/GN/GCE) [32], CeO_2_ nanoparticles dispersed in Brij^®^ 35 [33], coimmobilized carboxylated multi-walled carbon nanotubes and surfactants (MWCNT-COOH-SDS/GCE) [34], multi-walled carbon nanotubes (MWNTs) [35], Nafion/multi-walled carbon nanotubes (Nafion/MWCNT/GCE) [36], uniform Ag@C@Ag core–shell structured nanocomposites [37] and other single-walled carbon nanotubes screen-printed electrode (SWCNT-SPE) [38], screen-printed electrode modified with La_2_O_3_/Co_3_O_4_ nanocomposite [39], MnY nanozeolite modified carbon paste electrode (MnY/CPE) [40] and polyacrylamide embedded graphite molecular imprinted polymer electrode (PAM@G–MIP) [41]. The DPV technique was mainly used for THY and CAR determination, because it is characterized by the high sensitivity and good separation of the recorded peaks.

The voltammetric investigations were carried out in aqueous buffer solutions: phosphate [29,32,33,35,39,40,41], Britton-Robinson [26,34], acetate [27,28] or in alcohols [30,31,36].

The analysis of the literature data shows that anhydrous acetic acid (HAc) has not been applied to the determination of these compounds. It has several interesting properties, such as a relatively wide potential window, the ability to dissolve both hydrophobic organic compounds and their matrix, as well as the necessary supporting electrolyte. A low dielectric constant of acetic acid (ε = 6.2 at 25 °C [42]) causes the appearance of significant ohmic potential drops, *IR*. This problem can be overcome by the use of microelectrodes [43,44,45].

So far, there has been a lack of literature data on the use of microelectrodes for the determination of THY and CAR. Therefore, the aim of this work was to develop the first voltammetric method for the simultaneous determination of the total amount of thymol and carvacrol in herbal spices. For that purpose, a mixture of glacial acetic acid and acetonitrile (20%, *v*/*v*) was applied as a solvent.

## 2. Results and Discussion

### 2.1. Influence of the Electrode Material and Composition of Solution on THY and CAR Oxidation Curves

In view of the limited solubility of thymol and carvacrol in water. there was chosen glacial acetic acid containing acetonitrile (20%, *v*/*v*), with 0.1 mol L^−1^ NaClO_4_ as a supporting electrolyte. The presence of AN in the solutions tested diminished their viscosity and increased electrical permittivity which resulted in an increase in the voltammetric signals (data not shown). This environment is optimal for further measurements because it guarantees a wide potential window, especially in the anodic region.

**Figure 1:**
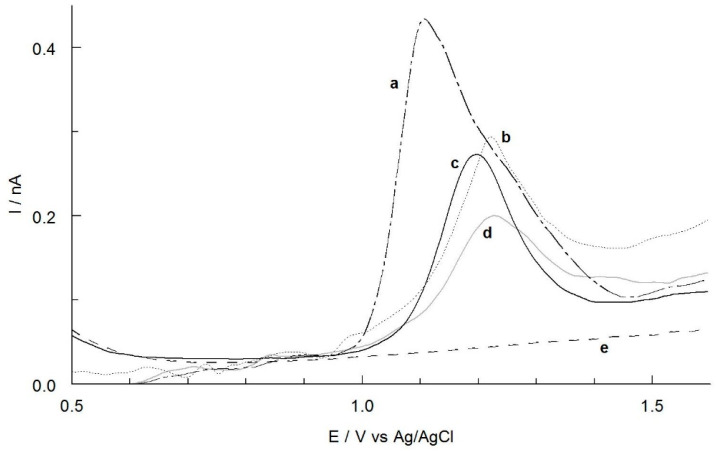
DPV curves of thymol (15 µg mL^−1^) oxidation recorded in solutions of HAc-AN (8:2, *v*/*v*) with 0.1 mol L^−1^ NaClO_4_ on: (a) gold, (b) carbon fiber, (c) platinum, (d) palladium microelectrode.

In order to optimize the measuring conditions, the influence of working electrode material was also studied. Investigations in the selected composition of the solution were carried out on palladium, gold, platinum, and carbon fiber (CF) microelectrodes (Figure 1). The best-shaped peaks of thymol and carvacrol oxidation and the lowest residual currents were obtained on the platinum microelectrode. This material provides the best reproducibility of the successively recorded curves. Therefore, a platinum disc microelectrode with a diameter of 25 μm was used in further studies.

### 2.2. Identification of THY and CAR

**Figure 2:**
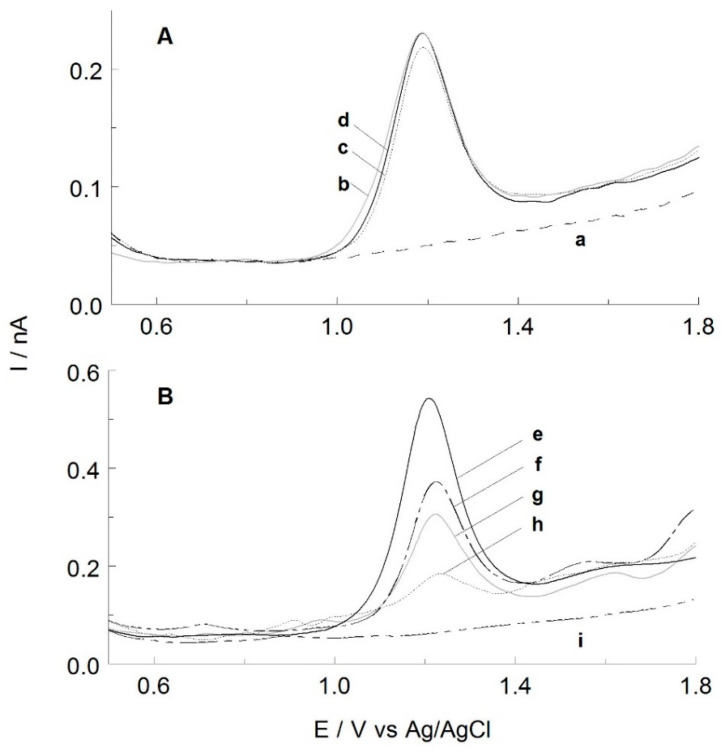
DPV oxidation curves recorded on a platinum microelectrode in solutions of (a) HAc-AN (8:2, *v*/*v*) with 0.1 mol L^−1^ NaClO_4_ containing (**A**) standards of: (b) THY, (c) CAR (each 10 µg mL^−1^) and (d) their mixture (each 5 µg mL^−1^), (**B**) spices (each 50 µL acetonitrile extract/mL): (e) oregano, (f) savory, (g) thyme (h) herbes de Provence and (i) rosemary.

Since thymol and carvacrol are geometric isomers, the curves recorded on the platinum microelectrode, in HAc-AN containing NaClO_4_, give well-formed peaks at a potential value of about 1.195 and 1.190 V vs. Ag/AgCl, respectively (Figure 2A). Owing to the difference of only 0.005 V between the peak potentials for THY and CAR, it is difficult to distinguish their voltammetric peaks. These results confirm the earlier observations taken from water solutions [28,46]. Therefore, only their total content can be voltammetrically determined.

Preliminary studies on the electrochemical properties of isopropylmethylphenols carried out on the Pt microelectrode using the LSV and DPV techniques showed that their electrode processes are quasi-reversible, controlled by diffusion and involves an exchange of one electron. The quasi-reversibility of the electron exchange reaction may increase the sensitivity of the determinations in comparison to the irreversible processes known from the literature [26,27,28,32,33,34,35,37,40,41].

### 2.3. Optimization of Extraction Condition

**Figure 3:**
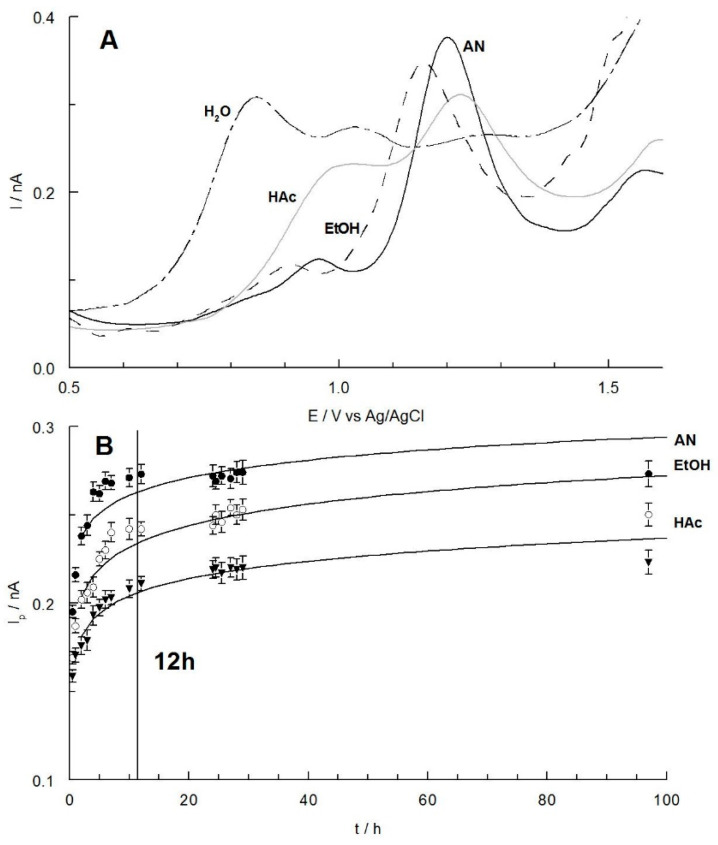
(**A**) Differential pulse voltammetry (DPV) curves of isopropylmethylphenols oxidation recorded in solutions containing thyme extracts (70 µL extract/mL, commercial spice) in selected solvents after 12 h of extraction. (**B**) Influence of the extraction time on the DPV peak current (*I*_p_) of the THY and CAR oxidation. Other components of the solutions as in Figure 2.

In the next stage of the study, the process of extracting analytes from real samples was optimized by selecting the appropriate solvent and extraction time. For that purpose, dried samples of thyme (0.5 g) were flooded with 10 mL of acetic acid, ethanol, or acetonitrile, respectively. Next, they were shaken mechanically. In case of water being used, infusions were prepared. Then, an appropriate volume of the extract or infusion was diluted with an experimentally chosen medium. The course of the DPV curves recorded in such prepared solutions (Figure 3A) shows that acetonitrile is the best solvent for extraction. The curves recorded in the solutions containing extract of HAc and EtOH and infusion are not well-shaped, asymmetrical and not reproducible.

In order to determine the optimal time of the extraction process, allowing the separation of the whole number of analytes from the plant matrix, DPV curves were recorded in solutions containing extracts taken after time ranging from 0.5 to 97 h. A successive increase in the peak current was observed, caused by the concentration of analytes in the extracts increasing with time (Figure 3B). This increase was greatest with extraction times of up to 10 h. After this time, the peak current gradually stabilized. This means that the concentration of THY and CAR in the extract reaches the maximum resulting from their content in the plant material. Based on the obtained results, it was found that the optimal extraction time of analytes from real samples should not be less than 12 h.

### 2.4. Identification of THY and CAR in Real Samples

In the next experiments, the voltammetric signal characteristic of isopropylmethylphenols, was identified in the solutions containing the extract samples of spices (Figure 2B). The peak corresponding to the oxidation of these analytes was observed at the potential of about 1.2 V vs. Ag/AgCl, which is close to this obtained for the mixture THY and CAR standard (Figure 2A, curve d). This means that the presence of the matrix did not modify position of the signal of isopropylmethylphenols oxidation on the potential axis. Noteworthy is the very good reproducibility of the successively recorded DPV curves in the real solution studied. This indicates that the electrode process is not accompanied by changes on the surface of the working electrode. Therefore, it is not necessary to polish it between experiments. This avoids errors related to loss of analyte during quantitative determinations.

### 2.5. Calibration Curves, Repeatability and Reproducibility

**Figure 4:**
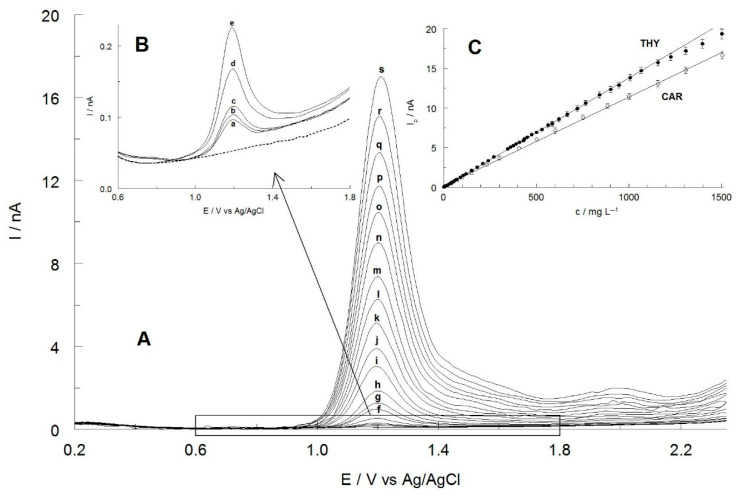
(**A**) DPV curves recorded in solutions containing different concentrations of CAR ranging from (a) 0.5 to (s) 1504 µg mL^−1^. (**B**) Enlarging part of the picture A. (**C**) Calibration plots for THY and CAR.

The calibration curves were constructed by measuring the peak current with optimized DPV parameters. Figure 4A,B display the DPV voltammograms at various concentrations of carvacrol. The identically shaped curves were obtained for THY (data not shown). The dependence of the peak current on thymol and carvacrol concentration shows wide linearity in the concentration range from 0.39 to 1105 and from 0.47 to 640 µg mL^−1^, respectively, as depicted in Figure 4C. The corresponding calibration equations are (*r* = 0.9999, for both): *I*_p_ (nA) = (0.01444 ± 5 × 10^−^^4^) *c* (µg mL^−1^) + (0.001809 ± 1.9 × 10^−^^4^) for THY, and *I*_p_ (nA) = (0.01202 ± 4 × 10^−^^4^) *c* (µg mL^−1^) + (0.01885 ± 2.1 × 10^−^^4^) for CAR. The detection limits (*LOD*) of thymol and carvacrol were calculated three times the value of the standard deviation of the intercept divided by the slope of the calibration curve and were 0.04 and 0.05 µg mL^−1^, respectively.

The comparison of the main analytical parameters characteristic of the methods determination of THY and CAR are presented in Table 1. The proposed procedure is characterized by the broadest linearity range and one of the lower detection limits. These parameters confirm the high sensitivity and utility of the developed method.

The intra-day repeatability was determined by the successive electrochemical measurements (*n* = 10) of thymol and carvacrol solutions (both 2 µg mL^−1^). The relative standard deviation of the peaks current of 1.2% (for THY) and 1.3% (for CAR), was obtained revealing excellent repeatability of the developed method. The inter-day repeatability of the results was determined based on measuring the peak currents for a period of 5 days with the use of the same solutions, and the obtained *RSD* values were 1.5% and 1.8%, respectively.

### 2.6. Interferences

Different compounds, such phenolic acids (chlorogenic, caffeic, rosmarinic), flavonoids (quercetin and rutin), citric and ascorbic acid, menthol, eugenol, glucose, sucrose, rhamnose, and also inorganic ions K^+^, Na^+^, Mg^2+^, Cu^2+^, Fe^2+^, SO_4_^2−^, Cl^−^ which are commonly present together with thymol and carvacrol in spices were tested as potential interferences. The most structurally related chemical compound to thymol and carvacrol are menthol and eugenol. Menthol is not electroactive in the range of potentials available in this environment. The impact of the 100-fold excess of interferences concentration on the peak currents of isopropylmethylphenols does not exceed 4%. These results indicate that it is possible to determine THY and CAR in the presence of these interferents.

### 2.7. Recovery Studies

**Figure 5:**
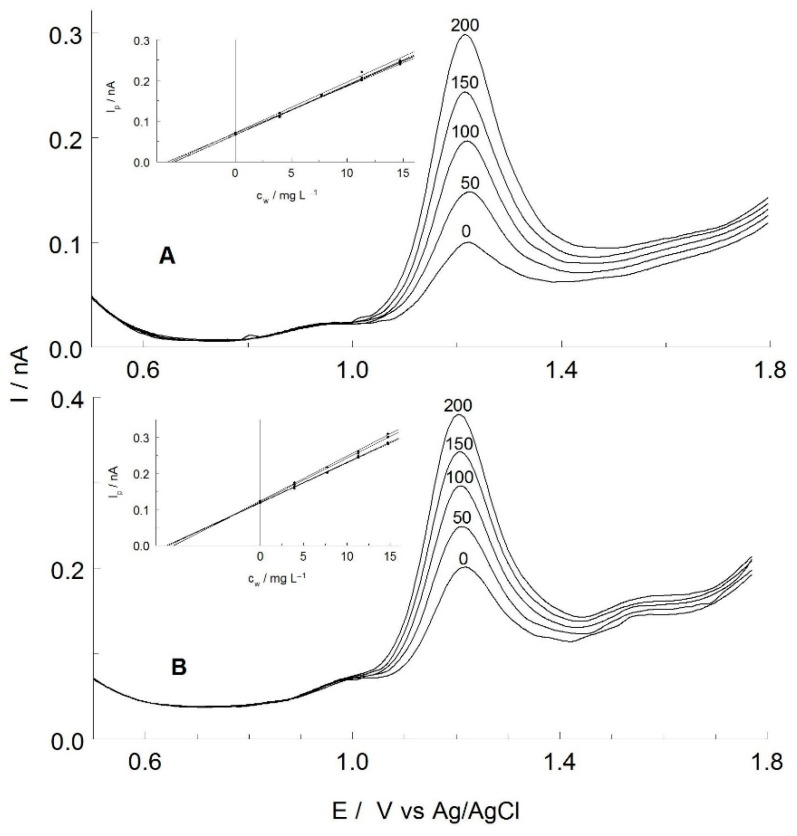
DPV curves recorded (**A**) in control solution of thymol (5.32 µg mL^−1^) with rosemary extract (30 µL/mL) and (**B**) in solution containing thyme extract (30 µL/mL) and after additions of THY standard solution (c = 162 µg mL^−1^, the volumes in μL are given at curves). Insets: calibration curves in the standard addition method for the control and spices sample, respectively.

To check the reliability and accuracy of the voltammetric method, a control determination was performed. For this purpose, a plant that does not contain the above-mentioned substances, i.e., rosemary, was used. A specified amount of THY was added to the rosemary in a sample, and then it was extracted with 10 mL of acetonitrile as, described above in Section 3.3. Next, 750 µL of the extract was taken into a 25 mL volumetric flask and mixed with the solvent solution used for analyses. 2.0 mL of this solution was transferred to an electrochemical cell, and DPV curves were recorded before and after the addition of the THY standard solution (Figure 5A). The determination was repeated five times. The concentrations of THY were converted per 1 g of the dry sample and were statistically examined (Table 2). The average content of thymol in the control solution only marginally different from the introduced amount (*R* = 101.0%), thus the developed method is accurate, and the relative standard deviation, *RSD* value of 0.3% indicates its high precision.

### 2.8. Real Sample Analysis

The developed method was applied for the determination of isopropylmethylphenols contents in spices. The preparation of the solutions tested was as previously described. There was a well-defined oxidation peak at 1.195 V on the DPV curves of thyme extracts (taken as an example) corresponding to the oxidation of isopropylmethylphenols, which was confirmed by using the standard addition method (Figure 5B). When the standard solution of THY was added to the measurement cell, a linear increase in oxidation currents occurred while maintaining the peak potential. It should be noted that all the curves recorded were very well reproducible. The inset in Figure 5B presents the calibration plots for five independent determinations. The isopropylmethylphenols contents in spice samples are presented in Table 2. The voltammetric data are agreed well with the results of an independent chromatographic determination. A comparison of the results using the Student’s *t*-test confirmed that both methods were equally accurate. (Table 2, *t*-values did not exceed critical ones). As can be seen, the precision of the results obtained by the voltammetric technique is comparable with those of GC (exceptions are Oregano and Herbes de Provence samples). It is to be noted that an important advantage of DPV method is the shorter measurement time.

## 3. Materials and Methods

### 3.1. Apparatus

The voltammetric experiments were carried out with a Model M161 electrochemical analyzer connected with the Model M162 preamplifier, co-operating with the software EALab 2.1. (mtm-anko, Cracow, Poland). The measurement cell consisted of a platinum microelectrode of 25 µm diameter (Mineral, Warsaw, Poland) as a working electrode, an Ag/AgCl (1 mol L^−1^ NaCl) reference electrode (Mineral, Warsaw, Poland), and a platinum wire as an auxiliary electrode (BASi, West Lafayette, IN, USA). The preliminary experiments were also performed on the microelectrodes made from platinum, gold, palladium (each 25 µm diameter, Mineral, Warsaw, Poland), and carbon fiber (CF, 33 µm diameter, BASi, West Lafayette, IN, USA). The surface of the working electrode was polished on a polishing cloth, with the use of 0.05 µm alumina powder (BASi, West Lafayette, IN, USA), rinsed with deionized water and dried before use. The grounded Faraday cage, in which the electrochemical cell was placed, prevented the influence of external interferences on the measured currents.

The GC measurements were carried out with the Model Thermo Scientific TRACE 1310 Gas Chromatograph (Thermo Fisher Scientific, Rodano, Italy) with a flame ionization detector (FID). The carrier gas was helium at a flow rate of 1 mL/min. The analytical Column TraceGOLD TG-5HT (30 m × 0.25 mm i.d., 0.25 µm f.t.) was used. The column temperature was programmed at 50 °C for 1 min. and then heated to 265 °C at a rate of 2.5 °C/min. The acquisition and processing of the data were performed using the Chromeleon™ Chromatography Data System (CDS, Thermo Fisher Scientific, Rodano, Italy) software version 7.

The extraction processes were performed using the orbital laboratory shaker Yellow Line OS 5 (IKA Werke, Staufen, Germany).

All the experiments were carried out at constant temperature (25 ± 1 °C).

### 3.2. Reagents and Materials

The chemicals used were as follows: thymol (THY), ≥99.5%, carvacrol (CAR), ≥98%, sodium perchlorate NaClO_4_, anhydrous, p.a. (all Sigma-Aldrich, St. Louis, MO, USA). Glacial acetic acid, CH_3_COOH (HAc), p.a. ACS and acetonitrile CH_3_CN (AN), p.a. (both Merck, Darmstadt, Germany) or their mixture were used as solvents in all electrochemical experiments. Acetonitrile with GC grade, purchased from VWR (Radnor, PA, USA), was used in the chromatographic analysis. All reagents and solvents were of high purity and used as received.

The samples of rosemary, thyme, oregano, savory, herbes de Provence (all Kamis S.A., Stefanowo, Poland) were in a dry form.

### 3.3. Electrochemical Measurements

For the qualitative and quantitative analysis of isopropylmethylphenols, differential pulse voltammetry (DPV) was selected. The optimized conditions for DPV technique were: potential step 5 mV, pulse width 80 ms, it is 2 × (t_waiting_ + t_sampling_), where t_waiting_ and t_samping_ were 20 ms, pulse amplitude 20 mV. The voltammetric measurements were performed on the platinum microelectrode in an experimentally chosen composition of the solution of glacial acetic acid containing 20% acetonitrile (*v*/*v*) and 0.1 mol L^−1^ sodium perchlorate as a supporting electrolyte.

The calibrations were based on DPV curves of THY and CAR oxidation on the platinum microelectrode in the concentration range from 0.5 to 1500 µg mL^−1^.

The determinations of isopropylmethylphenols in the test solutions and spices samples were conducted using a multiple standard addition method. Thymol in the concentration of about 160 µg mL^−1^, and with a volume of 50 µL was used as a standard solution. Its concentration was determined using the calibration curve.

**Scheme 2:**
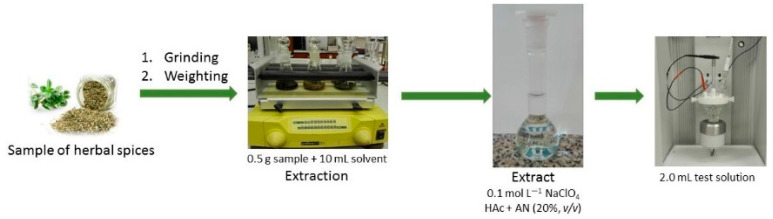
Flow chart of the course of experiments.

Commercially available samples of rosemary, thyme, oregano, savory, herbes de Provence were studied. They were first ground in an agate mortar. A representative portion of the milled samples was accurately weighted (0.5 ± 0.0001 g) into a 20-mL flask with a cap, and then 10 mL of solvent (ethanol, acetic acid or acetonitrile) was added. Next, the samples were shaken for a given time (up to 97 h) on a laboratory orbital shaker. In case of water being used, infusions were prepared. Then, the extracts were filtered through a 0.45 µm membrane filter before the voltammetric analysis (Scheme 2).

The test solutions of the spice samples were prepared in a 25 mL volumetric flask by diluting their acetonitrile extracts in an experimentally chosen medium and maintaining a constant concentration of NaClO_4_ and acetonitrile 0.1 mol L^−1^ and 20% (*v*/*v*), respectively. The volume of the parent solutions taken depended on the concentration of thymol and carvacrol in those samples. It was experimentally proved that the optimum peak current for their determination should be about 0.3 nA. All stock solutions were stored in a cool and dark place.

### 3.4. GC Measurements

Thymol and carvacrol were also determined using the reference GC method using a procedure proposed by Hajimehdipoor et al. [17] with some modifications. The stock solutions of thymol and carvacrol (about 2000 µg mL^−1^) were prepared in acetonitrile. Next, serial dilutions (20–200 µg mL^−1^) were made using the stock solutions. Calibration plots were constructed based on the THY and CAR peak areas versus concentration. These curves were used for the determination of isopropylmethylphenols in the spice samples. The procedure of extracting analytes from the spices was the same as for the voltammetric analysis. An appropriate amount of herbal extract was added to a volumetric flask and diluted to 5 mL with acetonitrile. This amount depended on the intensity of the signal in the chromatogram, which had to be within the linear range of the calibration curve. Each sample was injected into the GC instrument five times.

## 4. Conclusions

To the best of our knowledge, this is the first presentation of an electrochemical procedure to determine isopropylmethylphenols on a platinum microelectrode in glacial acetic acid containing acetonitrile (20%, *v*/*v*) and 0.1 mol L^−1^ sodium perchlorate as the supporting electrolyte. Differential pulse voltammetry was used to record the analytical signals. The calibration graphs were linear from 0.39 to 1105, and 0.47–640 µg mL^−1^, detection limits were 0.04 and 0.05 µg mL^−1^ for thymol and carvacrol, respectively. This method was successfully applied to the quantification of total thymol and carvacrol contents in spices using preliminary extraction with acetonitrile. The proposed conditions for the determination of isopropylmethylphenols prevented interferences with other matrix components of the spices. The GC analysis supported the reliability of the electroanalytical approach of the developed method.

The proposed method for the voltammetric determination of isopropylmethylphenols is characterized by high sensitivity, simplicity, reproducibility of results and can be recommended as an alternative method to control the quality of spices.

## Figures and Tables

**Table 1:** Comparison of the main analytical parameters characteristic of methods of thymol and carvacrol determination.

^1)^ Method	Analyte	LR/µg mL^−1^	LOD/µg mL^−1^	Reference
GC-MS	THY CAR	2 × 10^−3^–0.4 2 × 10^−3^–0.4	0.89 × 10^−3^ 0.57 × 10^−3^	[11]
GC-FID	THY	0.1–100	0.05	[13]
RP-LC-FLD	THY CAR	8 × 10^−3^–0.2 8 × 10^−3^–0.2	1.70 × 10^−3^ 1.56 × 10^−3^	[15]
RP-HPLC-UV	THY CAR	1–80 1–80	0.6 0.8	[16]
RP-HPLC-UV	THY	15–90	2.8	[17]
RP-HPLC-UV	THY CAR	15–75 1.5–7.5	0.17 × 10^−3^ 0.161 × 10^−3^	[18]
RP-HPLC-UV	THY	4–180	0.4	[19]
UV	THY	0.25–10	0.063	[24]
SWV (BDDE)	THY	0.6–15	0.59	[26]
DPV (BDDE)	CAR	0.29–15.23	0.02	[27]
DPV (BDDE)	THY CAR	0.34–9.52 0.29–11.56	0.02 0.02	[28]
SWV (GCE)	THY CAR	^2)^ nd	0.24 0.21	[29]
CV (GCE)	THY	9.01–300.4	1.50	[30]
LSV (GCE)	THY CAR	12.77–195.29 11.87–180.26	11.87 12.92	[31]
DPV (CeO_2_/GN/GCE)	THY	0.015–2.7	7.5 × 10^−3^	[32]
DPV (CeO_2_-Brij^®^ 35/GCE)	THY	0.10–91.03	0.03	[33]
LSV (MWCNT-COOH-SDS/GCE)	THY	0.075–22.53	0.021	[34]
DPV (MWNTs/GCE)	CAR	0.015–22.53	0.01	[35]
DPV (Nafion/MWCNT/GCE)	THY	0.075–0.750	7.5 × 10^−3^	[36]
DPV (Ag@C@Ag/GCE)	THY	0.015–1.50	3.2 × 10^−3^	[37]
DPV (SWCNT-SPE)	THY+CAR	nd	0.6	[38]
DPV (La_2_O_3_/Co_3_O_4_/SPE)	CAR	1.50–120.2	0.15	[39]
DPV (MnY/CPE)	THY	0.075–4.21	0.036	[40]
DPV (PAM@G-MIP)	THY	0.075–40.07	6.5 × 10^−3^	[41]

^1)^ symbols have been explained in the Introduction, ^2)^ no data.

**Table 2:** Results of the isopropylmethylphenols determination in control and in spices samples by DPV compared with a reference gas chromatography (GC) method.

Sample	DPV		GC		*t*-Test ^4)^ (2.31)	*F*-Test ^4)^ (6.39)
	^1)^ Amount Found/ mg g^−1^	^2)^*RSD*/% (*n* = 5)	^1)^ Amount Found/ mg g^−1^	^2)^*RSD*/% (*n* = 5)		
Control (rosemary matrix)	26.88 ± 0.11 ^3)^ (101.0%)	0.3	26.11 ± 0.41 ^3)^ (98.4%)	1.6	1.69	20.2
Thyme	6.91 ± 0.31	3.6	7.21 ± 0.30	3.3	0.86	1.09
Oregano	13.21 ± 0.04	0.3	13.33 ± 0.48	2.9	0.31	125.1
Savory	8.48 ± 0.15	1.4	8.16 ± 0.34	3.4	1.06	5.29
Herbes de Provence	2.15 ± 0.02	0.6	2.07 ± 0.04	1.7	2.12	7.50

^1)^ x = x_av_ ± t_0.95_ S_av_ for *n* = 5 and t_0.95_ = 2.776 (tabulated), x_av_—denote average value, S_av_—standard deviation of mean, ^2)^ relative standard deviation, ^3)^ Recovery, *R* = (x_av_/concentration involved) × 100%, ^4)^ values in parenthesis are tabulated *t* and *F* at *p* = 0.05, *n* = 5.

## Data Availability

The data presented in this study are available on request from the corresponding author.

## Sample Availability

Samples of thymol and carvacrol are available from the authors.

## References

[1] Scalbert A., Manach C., Morand C., Rémésy C., Jiménez L. (2005). Dietary polyphenols and the prevention of diseases. Crit. Rev. Food Sci. Nutr..

[2] Martins M.A.R., Silva L.P., Ferreira O., Schröder B., Coutinho J.A.P., Pinho S.P. (2017). Terpenes solubility in water and their environmental distribution. J. Mol. Liq..

[3] Breitmaier E. (2006). Terpenes—Flavors, Fragrances, Pharmaca, Pheromones.

[4] Cattelan M.G., de Nishiyama Y.P.O., Gonçalves T.M.V., Coelho A.R. (2018). Combined effects of oregano essential oil and salt on the growth of Escherichia coli in salad dressing. Food Microbiol..

[5] Fadil M., Fikri-Benbrahim K., Rachiq S., Ihssane B., Lebrazi S., Chraibi M., Haloui T., Farah A. (2018). Combined treatment of *Thymus vulgaris* L., *Rosmarinus officinalis* L. and *Myrtus communis* L. essential oils against *Salmonella typhimurium*: Optimization of antibacterial activity by mixture design methodology. Eur. J. Pharm. Biopharm..

[6] Starliper C.E., Ketola H.G., Noyes A.D., Schill W.B., Henson F.G., Chalupnicki M.A., Dittman D.E. (2015). An investigation of the bactericidal activity of selected essential oils to Aeromonas spp.. J. Adv. Res..

[7] Bertuola M., Fagali N., Fernández Lorenzo de Mele M. (2020). Detection of carvacrol in essential oils by electrochemical polymerization. Heliyon.

[8] Zarrini G., Delgosha Z.B., Moghaddam K.M., Shahverdi A.R. (2010). Post-antibacterial effect of thymol. Pharm. Biol..

[9] Ribeiro A.R.S., Diniz P.B.F., Pinheiro M.S., Albuquerque-Júnior R.L.C., Thomazzi S.M. (2016). Gastroprotective effects of thymol on acute and chronic ulcers in rats: The role of prostaglandins, ATP-sensitive K+ channels, and gastric mucus secretion. Chem. Biol. Interact..

[10] Tisserand R., Young R. (2014). Essential Oil Safety. A Guide for Health Care Professionals.

[11] Fiori G.M.L., Bonato P.S., Pereira M.P.M., Contini S.H.T., Pereira A.M.S. (2013). Determination of thymol and carvacrol in plasma and milk of dairy cows using solid-phase microextraction. J. Braz. Chem. Soc..

[12] Cacho J.I., Campillo N., Viñas P., Hernández-Córdoba M. (2015). Evaluation of three headspace sorptive extraction coatings for the determination of volatile terpenes in honey using gas chromatography-mass spectrometry. J. Chromatogr. A.

[13] Nozal M., Bernal J., Jiménez J., González M., Higes M. (2002). Extraction of thymol, eucalyptol, menthol, and camphor residues from honey and beeswax. J. Chromatogr. A.

[14] Kohlert C., Abel G., Schmid E., Veit M. (2002). Determination of thymol in human plasma by automated headspace solid-phase microextraction-gas chromatographic analysis. J. Chromatogr. B Anal. Technol. Biomed. Life Sci..

[15] Viñas P., Soler-Romera M.J., Hernández-Córdoba M. (2006). Liquid chromatographic determination of phenol, thymol and carvacrol in honey using fluorimetric detection. Talanta.

[16] Ghiasvand A., Dowlatshah S., Nouraei N., Heidari N., Yazdankhah F. (2015). A solid-phase microextraction platinized stainless steel fiber coated with a multiwalled carbon nanotube-polyaniline nanocomposite film for the extraction of thymol and carvacrol in medicinal plants and honey. J. Chromatogr. A.

[17] Hajimehdipoor H., Shekarchi M., Khanavi M., Adib N., Amri M. (2010). A validated high performance liquid chromatography method for the analysis of thymol and carvacrol in *Thymus vulgaris* L. volatile oil. Pharmacogn. Mag..

[18] Dedić M., Bečić E., Imamović B., Žiga N., Medanhodžić-Vuk S., Šober M. (2018). HPLC method for determination the content of thymol and carvacrol in Thyme tincture. Bull. Chem. Technol. Bosnia Herzegovina.

[19] Louchard B.O., Costa L.C., Silva A.R., Leal L.K.A. (2017). Validation of A High Performance Liquid Chromatography Method to Quantify Thymol in Nanocapsules of Bioactive Essential Oil from Lippia Sidoides. Int. J. Complement. Altern. Med..

[20] Zima J., Cienciala M., Barek J., Moreira J.C. (2007). Determination of thymol using HPLC-ED with glassy carbon paste electrode. Chem. Anal..

[21] Angelo T., Pires F.Q., Gelfuso G.M., da Silva J.K.R., Gratieri T., Cunha-Filho M.S.S. (2016). Development and validation of a selective HPLC-UV method for thymol determination in skin permeation experiments. J. Chromatogr. B Anal. Technol. Biomed. Life Sci..

[22] Martel A.C., Zeggane S. (2002). Determination of acaricides in honey by high-performance liquid chromatography with photodiode array detection. J. Chromatogr. A.

[23] Tonello N., D’Eramo F., Marioli J.M., Crevillen A.G., Escarpa A. (2018). Extraction-free colorimetric determination of thymol and carvacrol isomers in essential oils by pH-dependent formation of gold nanoparticles. Microchim. Acta.

[24] Fadhil G. (2014). Spectrophotometric Determination of Thymol in Pharmaceutical Preparations Via Oxidative Coupling Reaction with 2,4-dinitrophenylhydrazine in the Presence of Potassium Periodate. Iraqi J. Sci..

[25] Blanc R., González-Casado A., Navalón A., Vílchez J.L. (2000). On the estimate of blanks in differential pulse voltammetric techniques: Application to detection limits evaluation as recommended by IUPAC. Anal. Chim. Acta.

[26] Stanković D.M. (2015). Sensitive voltammetric determination of thymol in essential oil of Carum copticum seeds using boron-doped diamond electrode. Anal. Biochem..

[27] Kowalcze M., Jakubowska M. (2019). Voltammetric determination of carvacrol on Boron Doped Diamond Electrode. Anal. Chim. Acta.

[28] Kowalcze M., Jakubowska M. (2019). Multivariate approach in voltammetric identification and simultaneous determination of eugenol, carvacrol, and thymol on boron-doped diamond electrode. Mon. Chem..

[29] Tonello N., Moressi M.B., Robledo S.N., D’Eramo F., Marioli J.M. (2016). Square wave voltammetry with multivariate calibration tools for determination of eugenol, carvacrol and thymol in honey. Talanta.

[30] Ziyatdinova G.K., Budnikov H.C. (2014). Evaluation of the antioxidant properties of spices by cyclic voltammetry. J. Anal. Chem..

[31] Robledo S.N., Pierini G.D., Nieto C.H.D., Fernández H., Zon M.A. (2019). Development of an electrochemical method to determine phenolic monoterpenes in essential oils. Talanta.

[32] Zhao X., Du Y., Ye W., Lu D., Xia X., Wang C. (2013). Sensitive determination of thymol based on CeO2 nanoparticle–decorated graphene hybrid film. New J. Chem..

[33] Ziyatdinova G., Ziganshina E., Cong P.N., Budnikov H. (2017). Voltammetric Determination of Thymol in Oregano Using CeO_2_-Modified Electrode in Brij^®^ 35 Micellar Medium. Food Anal. Methods.

[34] Ziyatdinova G.K., Romashkina S.A., Ziganshina E.R., Budnikov H.C. (2018). Voltammetric Determinations of Thymol on an Electrode Modified by Coimmobilized Carboxylated Multiwalled Carbon Nanotubes and Surfactants. J. Anal. Chem..

[35] Ziyatdinova G., Budnikov H. (2021). MWNT-Based Electrode for the Voltammetric Quantification of Carvacrol. Food Anal. Methods.

[36] Piech R., Paczosa-Bator B. (2015). Application of glassy carbon electrode modified with Nafion/MWCNTs for sensitive voltammetric determination of thymol. Acta Pol. Pharm. Drug Res..

[37] Gan T., Lv Z., Deng Y., Sun J., Shi Z., Liu Y. (2015). Facile synthesis of monodisperse Ag@C@Ag core–double shell spheres for application in the sensing of thymol and phenol simultaneous. New J. Chem..

[38] Fuentes F.G., Gil M.Á.L., Mendoza S., Escarpa A. (2011). Electrochemical screening of biomarkers in chemotype Mexican oregano oils on single-walled carbon nanotubes screen-printed electrodes. Electroanalysis.

[39] Mohammadi S.Z., Beitollahi H., Rohani T., Allahabadi H. (2019). Carvacrol electrochemical reaction characteristics on screen printed electrode modified with La_2_O_3_/Co_3_O_4_ nanocomposite. J. Electrochem. Sci. Eng..

[40] Aghamohseni B., Hassaninejad-Darzi S.K., Asadollahi-Baboli M. (2019). A new sensitive voltammetric determination of thymol based on MnY nanozeolite modified carbon paste electrode using response surface methodology. Microchem. J..

[41] Naskar H., Biswas S., Tudu B., Bandyopadhyay R., Pramanik P. (2019). Voltammetric Detection of Thymol (THY) Using Polyacrylamide Embedded Graphite Molecular Imprinted Polymer (PAM@G-MIP) Electrode. IEEE Sens. J..

[42] Izutsu K. (2009). Electrochemistry in Nonaqueous Solutions.

[43] Bond A.M. (1994). Past, present and future contributions of microelectrodes to analytical studies employing voltammetric detection. A review. Analyst.

[44] Stojek Z. (1991). New possibilities in analytical chemistry connected with voltammetric applications of microelectrodes. Mikrochim. Acta.

[45] Michalkiewicz S., Tutaj M., Kaczor M., Malyszko J. (2002). Electrochemical behavior of tocopherols on microelectrodes in acetic acid medium. Electroanalysis.

[46] Michelitsch A., Rittmannsberger A., Hüfner A., Rückert U., Likussar W. (2004). Determination of isopropylmethylphenols in black seed oil by differential pulse voltammetry. Phytochem. Anal..

